# An [18F]FDG-PET/CT deep learning method for fully automated detection of pathological mediastinal lymph nodes in lung cancer patients

**DOI:** 10.1007/s00259-021-05513-x

**Published:** 2021-09-14

**Authors:** David Wallis, Michaël Soussan, Maxime Lacroix, Pia Akl, Clément Duboucher, Irène Buvat

**Affiliations:** 1 Laboratoire D’Imagerie Translationnelle en Oncologie, U1288 Inserm, Institut Curie, PSL, Université Paris Saclay, Paris, France; 2 Department of Nuclear Medicine, Avicenne Hospital, APHP, Bobigny, Paris France

**Keywords:** Deep learning, Lung cancer, Automated analysis, Lymph nodes, PET/CT

## Abstract

**Purpose:**

The identification of pathological mediastinal lymph nodes is an important step in the staging of lung cancer, with the presence of metastases significantly affecting survival rates. Nodes are currently identified by a physician, but this process is time-consuming and prone to errors. In this paper, we investigate the use of artificial intelligence–based methods to increase the accuracy and consistency of this process.

**Methods:**

Whole-body ^18^F-labelled fluoro-2-deoxyglucose ([18F]FDG) positron emission tomography/computed tomography ([18F]FDG-PET/CT) scans (Philips Gemini TF) from 134 patients were retrospectively analysed. The thorax was automatically located, and then slices were fed into a U-Net to identify candidate regions. These regions were split into overlapping 3D cubes, which were individually predicted as positive or negative using a 3D CNN. From these predictions, pathological mediastinal nodes could be identified. A second cohort of 71 patients was then acquired from a different, newer scanner (GE Discovery MI), and the performance of the model on this dataset was tested with and without transfer learning.

**Results:**

On the test set from the first scanner, our model achieved a sensitivity of 0.87 (95% confidence intervals [0.74, 0.94]) with 0.41 [0.22, 0.71] false positives/patient. This was comparable to the performance of an expert. Without transfer learning, on the test set from the second scanner, the corresponding results were 0.53 [0.35, 0.70] and 0.24 [0.10, 0.49], respectively. With transfer learning, these metrics were 0.88 [0.73, 0.97] and 0.69 [0.43, 1.04], respectively.

**Conclusion:**

Model performance was comparable to that of an expert on data from the same scanner. With transfer learning, the model can be applied to data from a different scanner. To our knowledge it is the first study of its kind to go directly from whole-body [18F]FDG-PET/CT scans to pathological mediastinal lymph node localisation.

**Supplementary Information:**

The online version contains supplementary material available at 10.1007/s00259-021-05513-x.

## Introduction

Lung cancer is the most commonly diagnosed cancer and the leading cause of cancer death worldwide, with non–small-cell lung cancer (NSCLC) comprising more than 85% of these cases [1, 2]. NSCLC typically metastasises to the hilar and mediastinal lymph nodes, and the presence of metastases significantly impacts the staging, prognosis, and patient management. Five-year survival rates are 54% for patients without any metastases and 27% for patients with mediastinal metastases [3]. Tumour progression, prognosis evaluation, and decisions on treatment plans are mainly dependent on this staging, and thus, it is critically important to accurately detect the mediastinal cancer nodes.

Currently, a ^18^F-labelled fluoro-2-deoxyglucose ([18F]FDG) positron emission tomography/computed tomography ([18F]FDG-PET/CT) scan is acquired, and the nuclear medicine physician/radiologist examine all the slices. The sensitivity and specificity of these lesion-based analyses have been shown to be 0.59 and 0.97, respectively, meaning many nodes remain undetected [4]. Additionally, the agreement between observers is limited. Inter-observer agreement, defined by the kappa score (*κ*), has been shown to range from 0.48 to 0.88, depending on the type of node (agreement was lower for aortopulmonary nodes (*κ* = 0.48–0.55) but higher for inferior and superior nodes (*κ* = 0.71–0.88)) [5]. We hypothesise that an artificial intelligence–based system could improve the sensitivity and reproducibility of mediastinal lymph node staging while also saving radiologists time.

In recent years, the use of machine learning algorithms to aid and automate medical-related problems has increased massively. Programmes have been built to automate tasks across the gamut of clinical oncology, from tumour detection and segmentation to therapy decisions [6–9]. A subset of machine learning, convolutional neural networks (CNNs) have emerged as a leading tool for classifying images [10]. In contrast to mathematical radiomic features, CNNs self-learn optimised features through a series of layers. Successive layers learn increasingly higher-level features from the images using non-linear mappings, eventually using these features to make predictions. The network is trained so that the features become optimised for the task. CNNs have shown promise in a wide range of image recognition and classification tasks, in some cases challenging the accuracy of medical experts [11–13]. One important issue impeding the widespread adoption of machine learning models is their lack of ability to generalise to data from different sources (*domain shift*) [14, 15]. A solution is to use transfer learning, which involves further training of the model using data from the second domain, often with some CNN layers frozen or with a smaller learning rate (also called fine-tuning). This has been shown to improve inter-scanner performance [16, 17].

For lung cancer, machine learning models have been built to detect pulmonary nodules in CT scans at an accuracy comparable to or better than that of physicians [18, 19]. Ardila et al. used a cohort of 14,851 patients from the publicly available National Lung Screening Trial to predict risk of lung cancer, achieving an AUC of 0.94 and outperforming radiologists [20].

However, there has been less progress on the automated detection of pathological mediastinal lymph nodes. Roth et al. used a CNN to automatically detect enlarged lymph nodes (indicating disease) from CT scans [21]. The input data consisted of 2.5D patches (three orthogonal slices) centred on the lymph node locations. With a cohort of 86 patients, they achieved a sensitivity of 0.70 with three false positives/patient.

Several [18F]FDG-PET thresholding methods have also been proposed, as described in the meta-analysis by Schmidt-Hansen et al. [22]. These studies used features such as [18F]FDG-PET SUV_max_ and [18F]FDG-PET SUV_mean_ to classify nodes as pathological. Using an *SUV*_*max*_ ≥ *2.5* criterion gave an average sensitivity and specificity of 0.81 and 0.79, respectively. However, the meta-analysis showed high inter-study heterogeneity. Additionally, these studies all required the nodes to first be located manually by an expert.

Given the diversity of mediastinal node shapes, sizes, and locations, using solely the CT or [18F]FDG-PET is limiting. Wu et al. showed that using [18F]FDG-PET/CT for nodal staging of NSCLC confers significantly higher sensitivity and specificity than only contrast-enhanced CT and higher sensitivity than only [18F]FDG-PET [23]. With a cohort of 168 patients, Wang et al. used a 2.5D CNN (using six axial patches of size 51 × 51 mm^2^) to diagnose mediastinal lymph node metastasis, achieving a sensitivity and specificity of 0.84 and 0.88, respectively [24].

While these models achieved good results, they all required as inputs the locations of the mediastinal nodes. Locating these nodes is already a time-consuming task. Furthering these studies, our aim was to build a model that could automatically classify mediastinal nodes directly from whole-body [18F]FDG-PET/CT scans, without the need for prior annotation. This is akin to other works that have used CNNs to go directly from whole-body [18F]FDG-PET/CT scans to suspicious [18F]FDG-PET/CT foci localisation [25, 26]. This model could then be used by a physician to quickly identify high-risk lymph nodes, saving time and increasing diagnosis consistency.

## Materials and methods

To achieve our goal, we used supervised learning to build a fully automated 3D end-to-end algorithm that used whole-body [18F]FDG-PET/CT scans as inputs and output the same scans with the locations of suspicious mediastinal nodes highlighted. The performance of the algorithm with respect to an experienced “reference” nuclear medicine physician was compared to that of a second physician with respect to the reference physician. The model was also tested on data from a different scanner with and without transfer learning.

### Datasets

Details of the datasets are shown in Table 1. An initial cohort of 134 NSCLC patients who underwent [18F]FDG-PET/CT scans (Gemini TF; Philips Medical Systems, Best, the Netherlands) was studied. This study was approved by an institutional review board. [18F]FDG-PET/CT was performed 60 min after intravenous injection of 3 MBq/kg of [18F]FDG, with 105-s acquisition per bed position. CT images were obtained without a contrast medium. Images were reconstructed using a blob ordered subset–time of flight list-mode iterative algorithm (2 iterations, 33 subsets, including attenuation and scatter corrections). [18F]FDG-PET voxels were 4 × 4 × 4 mm^3^. CT in-plane resolution varied from 0.58 mm to 1.05 mm, and the inter-plane resolution was 1 mm. All scans were analysed under the supervision of an experienced dual board–certified radiologist (15 years’ experience in thoracic imaging), who used the LIFEx [27] software to mark the positions of mediastinal nodes they would consider positive in routine clinical examination. These positions served as a surrogate ground truth. Nine patients were excluded from the study because of multimetastatic disease involving the lung, pleura, and the mediastinum leading to unreliable nodal identification. This gave a total of 125 patients comprising 172 positive nodes. Twenty-nine randomly chosen patients (comprising 52 positive nodes) were reserved for testing and only used for the final evaluation of the model. For comparison of the variation between our algorithm and the physician and that between two physicians, this test set was also labelled independently by a second physician (5 years’ experience in thoracic imaging). For hyperparameter optimisation, a validation set of 24 patients comprising 35 nodes was used. During final evaluation of the model, the validation set was included in the training set.

**Table 1:** Summary of the datasets, showing the numbers of patients and positive nodes in each cohort. Also shown are the distributions of patients by number of nodes per patient

		Scanner 1		Scanner 2	
		Training set	Test set	Training set	Test set
Number of patients		96	29	30	29
Number of nodes		120	52	35	34
Patients split by number of nodes	0	55	12	13	16
	1	11	6	6	3
	2	13	3	7	3
	3	6	1	2	4
	4 +	11	7	2	3

**Fig. 1:**
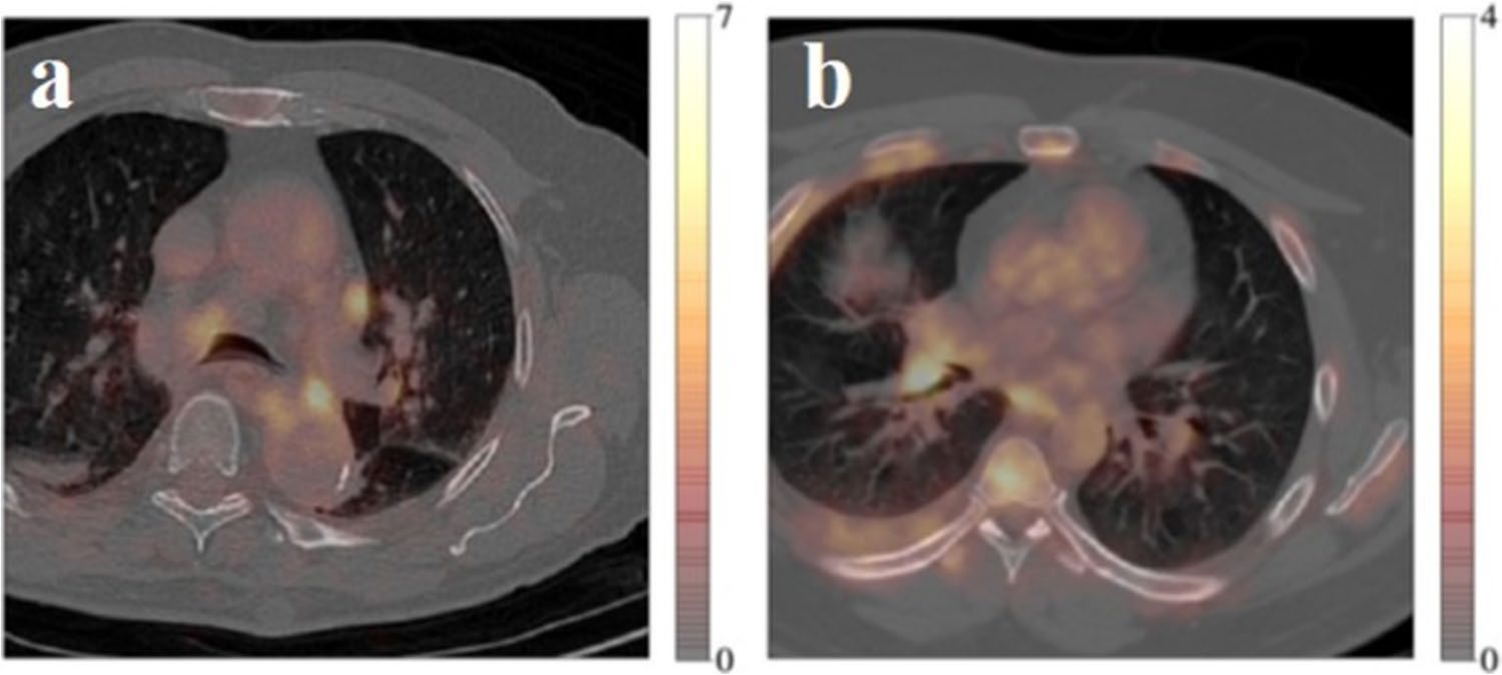
A comparison of [18F]FDG-PET/CT images from **a** scanner 1, Gemini TF (Philips Medical Systems), and **b** scanner 2, Discovery MI (GE Medical Systems)

A second cohort of 71 patients was later acquired on a different scanner from the same institution. These data were acquired on a digital GE Discovery MI PET/CT (DMI) system with a 25-cm FOV. PET scans were performed around 60 min after intravenous injection of 2.5 MBq/kg of [18F]FDG, with 120-s acquisition per bed position. Data were reconstructed using BSREM (Q.Clear) with a penalisation β-factor set at 800. [18F]FDG-PET voxels were of size 2.8 mm inter-plane and varied from 2.34 to 2.73 mm in-plane. CT in-plane resolution varied from 0.8 to 1.37 mm, and inter-plane resolution was 1.25 mm. These scans, from a newer scanner, had improved contrast (see Fig. 1 for a comparison). Positions of pathological mediastinal nodes were marked by the same expert to serve as a surrogate ground truth. Twelve patients were excluded due to the reasons above, giving a total of 59 patients comprising 69 positive nodes. From these, 30 patients (comprising 35 nodes) were reserved for transfer learning training, leaving 29 patients (comprising 34 nodes) for testing.

### Automated image analysis

**Fig. 2:**
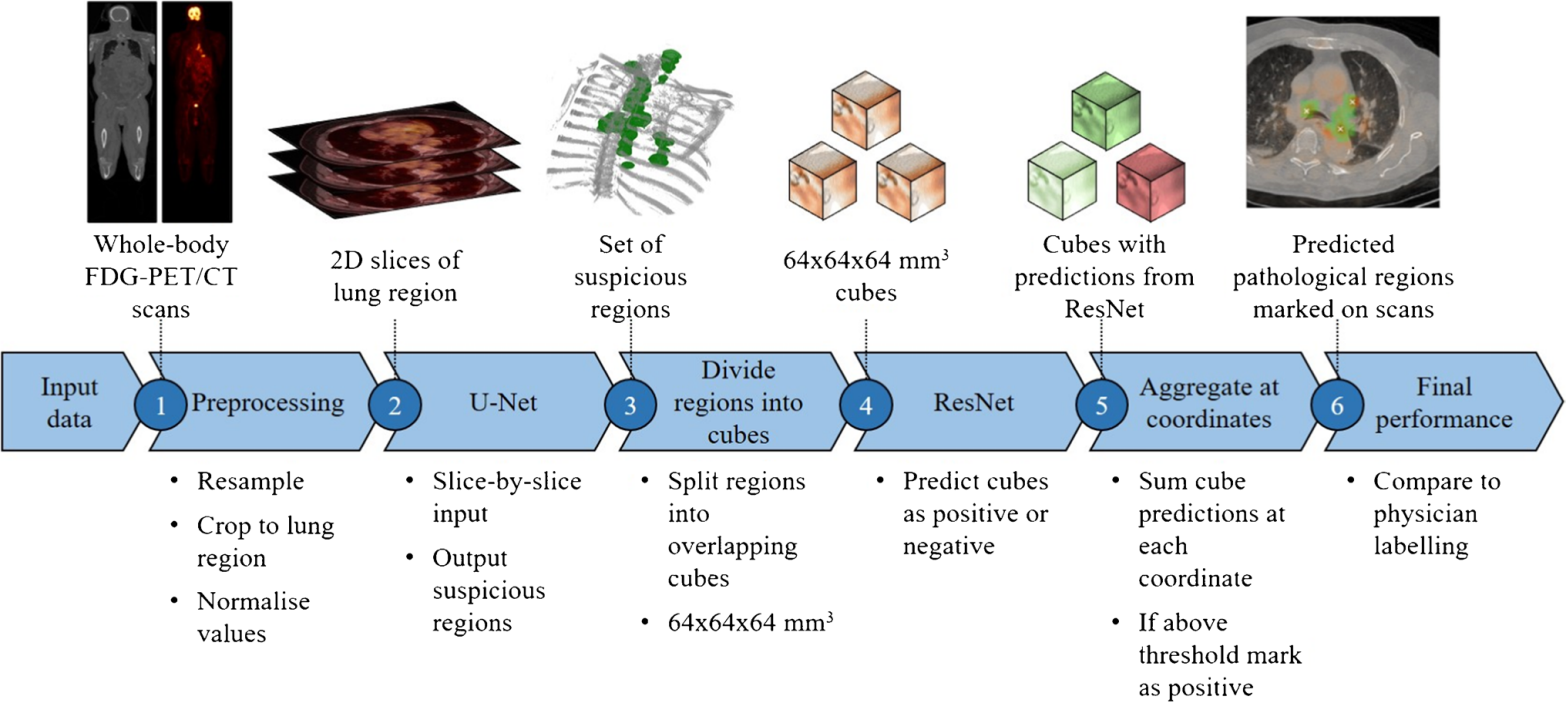
Process diagram showing the model pipeline. Images are first preprocessed to give slices only in the thorax. These are then fed into a U-Net to give a set of candidate regions. These are then divided into overlapping cubes, which are input into a 3D ResNet for classification. This gives a set of predictions at each coordinate, which are aggregated to give the final predicted pathological nodes

The proposed pipeline for the model is shown in Fig. 2 and consists of several stages, starting with the whole-body [18F]FDG-PET/CT images and ending with a list of predicted pathological node locations. All experiments were run on a single Nvidia GeForce RTX 2080Ti graphics card. CNNs were built using Keras 2.4.3 with a TensorFlow 2.2.2 backend.

#### Data preprocessing

Both the [18F]FDG-PET and CT scans were resampled to give images with uniform voxels of size 1 × 1 × 1 mm^3^ using a cubic spline interpolation. The raw DICOMs were whole-body scans, but only the thorax was relevant. This region was isolated using a crude lung segmentation algorithm, based on [28]. The CT scan was first thresholded, so any voxel with a Hounsfield unit (HU) greater than –400 was set to 0 and anything below set to 1. This identified the air-filled regions of the CT. This was followed by a two-iteration binary closing operation and a seven-iteration binary opening operation (both with a square connectivity of one). These operations removed very small air-filled regions and removed small nodules within the lungs. Any air regions outside the patient were discounted (these were automatically identified as any regions within 25 voxels of the image border). The largest air-filled region was then selected, in all cases one or both lungs. To automatically identify the thorax, only slices within the range of this segmentation were kept, and the whole-body scans were cropped to leave just slices within these bounds. Slices were then symmetrically cropped to size 256 × 256 pixels to remove large non-relevant regions outside the body. CT values were bounded such that any values above/below –1000/1000 HU were set to –1000/1000, and [18F]FDG-PET values above 8 SUV were set to 8 SUV. [18F]FDG-PET and CT values were finally rescaled to give the dataset zero mean and unit variance, using the whole training data to calibrate the rescaling (this dataset-level rescaling ensured that absolute differences in values between patients were maintained). This data preprocessing was fully automated and did not require any manual supervision.

#### Classification phase one: generate candidates

The initial dataset was very unbalanced, with only a small number of positive mediastinal nodes compared to the large total image volume. Because of this, a two-phase process was used for classification.

Phase one was a “candidate generation” step, with the goal of producing a small set of candidate pathological regions that retained most of the true positive nodes (high sensitivity). To do this, a 2D U-Net was trained [29]. The mediastinal nodes were not explicitly segmented, so simple spheres of radius 15 mm centred on the nodes were used as labels (most nodes are of diameter 0–30 mm, so this ensured that the entire nodes were within the labelled regions [30]). The U-Net was trained on a slice-by-slice basis, with the training data being 256 × 256 × 2 pixel [18F]FDG-PET/CT slices (full details of the CNN architecture can be found in Supplementary Material 1) and the output being the positive node regions. Only slices containing positive labels were used in training. This whole-slice view was used so the network had a full physiological view of each slice and could learn to only pick candidates within the mediastinum. The network was trained for 20 epochs with a Dice loss and an Adam optimiser with a learning rate of 10^–5^, by which time the validation loss had stopped decreasing.

Once trained, for each patient, the entire 3D volume was input into the U-Net slice-by-slice, and a set of contiguous candidate regions was produced. Any region with a volume of less than 300 voxels was removed, as these were clearly too small to be node candidates. A three-iteration dilation was then performed (with a square connectivity of one) to expand the candidate regions, ensuring that the entirety of each node was covered by the regions.

#### Classification phase two: false positive reduction

Phase two classified the regions from phase one as negative or positive, i.e. determined if they included a cancerous mediastinal node. In some cases, the regions were large, meaning just predicting the entire region as pathological would not have been useful, as the location of the pathological node would not have been precisely identified. Therefore, the regions were split into overlapping 3D cubes at 8-mm intervals in the *x*, *y*, and *z* axes (forming a regular grid). The cubes were of side length 64 mm.

This gave a total of 46,427 cubes in the training set. Any cubes with a pathological node within 16 mm of their centres were marked as positive, and those without were marked as negative (giving 4,235 positive cubes and 42,192 negative cubes). During training, the negative cubes were randomly undersampled by a factor eight. This considerably sped up training with a negligible effect on performance.

A 50-layer 3D ResNet architecture was used to classify the cubes, based on [31]. The architecture and code for this model are available at [32]. The network was trained for 20 epochs with a batch size of 32 using an Adam optimiser with a learning rate of 10^–5^ and a cross-entropy loss, by which point the validation loss had stopped decreasing. On-the-fly augmentation was used in the form of random rotations and flips in the *x*, *y*, and *z* axes.

Once trained, this network was used to give a pathological probability for each cube. As the cubes overlapped, there were several predictions for each coordinate. To aggregate these, the predictions at each coordinate were summed, and any coordinate above a threshold of 18 was considered positive (this threshold was found by minimising the sum of the false positives and false negatives on the validation set).

#### Performance assessment

This map of pathological locations was useful for viewing the locations of pathological nodes, but to compare it directly with the accuracy of physicians and with other studies, it needed to be converted to an appropriate metric, as the accuracy of cube classification is not equivalent to the accuracy of node classification.

To determine the node classification accuracy, each contiguous region that had been predicted positive was labelled. Any regions that had pathological nodes within them were classed as true positives (TPs), any without nodes were classed as false positives (FPs), and any pathological nodes that were not contained in a region were classed as false negatives (FNs). Using this measure, one large region spanning the whole scan would constitute perfect accuracy despite being completely useless, so the regions were visually checked to ensure that they were small enough to localise the nodes. These three numbers were used to calculate the sensitivity and FPs/patient. These metrics are sufficient to give a full performance analysis of the model (the key points being numbers of false positives and false negatives). Previous works mentioned above use specificity as a benchmark, although there is no consistency in the number of nodes per patient (the CNN analysis in [24] had an average of 8.3 nodes per patient, whereas in a meta-analysis of physician performance [4], the number of nodes per patient varied from 0.8 to 12.7). We therefore did not use specificity as a performance metric.

The performance was first assessed using the test set of 29 patients from the same scanner. During training, our “ground truth” was the labelling by an experienced physician, but as mentioned in the introduction, this labelling is not perfect. Therefore, a more informative metric is the comparison of the variation of our algorithm with respect to a physician to the variation of one physician with respect to another. Using the labelling by the second physician, inter-observer agreement was measured using the kappa statistic [33], using the benchmarks of Landis and Koch [34] (0.81–1, almost perfect agreement; 0.61–0.8, substantial agreement; 0.41–0.6, moderate agreement; 0.21–0.4, fair agreement). Calculation of the kappa statistic requires the number of true negatives. This was determined by subtracting the sum of TPs, FPs, and FNs from 8.3 multiplied by the number of patients. This was for consistency with [24], where the analysis had 8.3 nodes per patient. Exact 95% Clopper-Pearson confidence intervals (CIs) were calculated for each performance metric [35].

#### Using data from the second scanner

Finally, the ability of the model to classify images from the second scanner was tested. The scanner 2 test set was first evaluated directly on the trained model. To see if this performance could be improved, the model was then fine-tuned using transfer learning. After generating a set of cubes using the exact same U-Net from phase one, the ResNet model from phase two was trained for 20 more epochs, using the training data from scanner 2 (the U-Net model was not fine-tuned). The training parameters were identical to those in phase two, but with a smaller learning rate of 10^–8^. This model was evaluated as above on the scanner 2 test data. This performance was compared to the performance without transfer learning.

## Results

After phase one, the model achieved 100% sensitivity on the test set from the first scanner (i.e. all positive nodes were detected). On the test set from the second scanner, two positive nodes were missed in phase one (94% sensitivity). Overall model results are detailed in Table 2.

**Table 2:** Summary of results for both test sets with and without model fine-tuning. 95% confidence intervals are shown

Experiment	Sensitivity	False positives/patient
Scanner 1	0.87 [0.74, 0.94]	0.41 [0.22, 0.71]
Scanner 2 (no transfer learning)	0.53 [0.35, 0.70]	0.24 [0.10, 0.49]
Scanner 2 (with transfer learning)	0.88 [0.73, 0.97]	0.69 [0.43, 1.04]

### Scanner 1

The results for scanner 1 correspond to a substantial agreement between the physician and our model (*κ* = 0.77 [95% CI 0.68, 0.87]). The agreement between the two physicians was also substantial (*κ* = 0.66 [0.54, 0.77]), as was that between our model and the second physician (*κ* = 0.71 [0.60, 0.82]). These results show that the agreement between our model and the physician is comparable to that between the two physicians.

Supplementary Fig. 1 shows some examples of the output of the model. These show how the model highlights regions it has predicted positive. These could aid a physician by quickly pointing them to suspicious areas, as well as drawing attention to suspicious areas they may have missed. Figures 1a and 1b show nodes correctly identified by the model. Figure 1c shows a false positive, where the model has marked a left thyroid nodule. Figure 1d shows another false positive, where the model has marked a region of inflammation next to the oesophagus. Figures 1e and 1f show two false negative cases. Both of these nodes show low uptake on the [18F]FDG-PET and do not stand out well from the background. These were marked by the physician because the sizes of the nodes are > 10 mm and the primary tumour is on the same side of the lungs as the nodes.

### Scanner 2

The performances on the scanner 2 test data with and without transfer learning are shown in Table 2. Applying our model directly to data from the second scanner resulted in a much lower sensitivity (0.53 [0.35, 0.70] vs 0.87 [0.74, 0.94]), indicating many more false negative cases. After using transfer learning to fine-tune the model, the sensitivity improved significantly, to 0.88 [0.73, 0.97]. However, the number of FPs/patient increased (0.24 [0.10, 0.49] vs 0.69 [0.43, 1.04]).

## Discussion

The performance of our model on the first test set was good, better than the reported performance of physicians (a meta-analysis of physician performance showed sensitivity and specificity values of 0.59 and 0.97, respectively, for lesion-based labelling [4]). However, without having a ground truth, these results are not directly comparable. Additionally, this meta-study showed a huge range in these results (sensitivity from 0.46 to 0.90 and specificity from 0.65 to 0.98). One factor that was shown to affect the results was geography (some studies were in high tuberculosis-prevalent regions, which lead to more false positives), but other factors such as scanner type, centre protocols, and physician experience may have made a difference. This variation highlights the difficulty in comparing results across studies, so any comparison must be treated with care. Taking this into account, our model achieved a comparable sensitivity to previous automated mediastinal node detection studies, with the CNN used in [24] achieving a sensitivity of 0.84. Most importantly, unlike all previous reports regarding the identification of mediastinal cancer nodes in lung cancer patients, our approach only requires the full [18F]FDG-PET/CT scan as an input and does not rely on preliminary manual identification of patches containing suspicious uptake, thus representing a significant improvement in the overall automation of the process. This complete automation increases the consistency and reproducibility of mediastinal lymph node labelling. As demonstrated in Supplementary Fig. 1, the model could thus be used to aid physicians by quickly guiding them to potential positive node sites.

Comparing the results to those between two physicians, the agreement between the algorithm and the first physician was similar to that between the two physicians (*κ* = 0.77 [0.68, 0.87] vs *κ* = 0.66 [0.54, 0.77]). The agreement between our model and the second physician was slightly lower (*κ* = 0.71 [0.60, 0.82]). This is not surprising, as our model was trained using the first physician’s labelling. Only one study could be found reporting inter-physician agreement in identifying pathological mediastinal nodes using [18F]FDG-PET/CT scans and reported an inter-observer agreement ranging from 0.48 to 0.88, depending on the type of mediastinal node [5]. These results are consistent with ours. As mentioned, however, comparisons to other studies should be treated with caution due to the potentially large variations.

When applying our model directly to data from the second scanner, the detection sensitivity dropped (Table 2). More work would be needed to determine which parameters are most relevant to this performance drop (scanner hardware, reconstruction parameters, etc.). Fine-tuning the model using transfer learning made it possible to improve the sensitivity up to that observed on the scanner 1 test data, but at the cost of more FPs/patient.

As is usually the case with CNNs, there is no sure-fire way to predict which parameters will result in the best performance, so a range of hyperparameter combinations were tested to optimise the model. In addition, single-phase approaches were tested (going directly from the scans to node locations) but found to not be discriminant enough, leaving too many false positives. Models with [18F]FDG-PET–only and CT-only inputs were also tested, but these produced poor results. This is probably because their uses are complimentary: the CT provides anatomical information to locate the nodes, and the PET provides metabolic information to determine if the nodes are positive. Full details of the parameters tested for all phases of model creation are summarised in the Supplementary Material.

One limitation of this study, and thus problem with the above comparisons, was the lack of comparison with a gold standard. Not all pathological nodes are detectable on [18F]FDG-PET/CT scans (e.g. [18F]FDG-negative cases), so without histological analysis, it is impossible to know which nodes are truly positives. However, histological analysis also has its limitations in this application. In standard clinical practice, only suspicious nodes are biopsied, and if a patient has several suspicious nodes, not all will be biopsied. Additionally, some nodal stations are easier to access and thus more often biopsied, leading to sampling errors. These problems may explain the huge range in the number of nodes per patient seen in the meta-analysis [4]. A consensus judgement would have improved our study, making our model less physician-specific.

Our dataset was quite small, and more data would undoubtedly improve these results. In a broader sense, this is the key advantage of a machine learning approach. As clinical practice adapts and training datasets get bigger, a CNN can learn from a broader range of nodes, allowing it to become more accurate. It could be that a certain misclassified node had no direct comparison in the training set, but with a larger training set, this would rarely happen.

## Conclusion

These results show a fully automated 3D CNN-based algorithm that can detect pathological mediastinal nodes with an inter-reader kappa score similar to that between two physicians. To our knowledge, it is the first study of its kind to go directly from whole-body [18F]FDG-PET/CT scans to pathological mediastinal lymph node predictions and locations in lung cancer patients. The results also show that with transfer learning, the model can be adapted to perform well on a test set from a different scanner. With more comprehensive testing (e.g. using a consensus-based judgement) and a larger dataset, we believe that this approach could be used to improve the sensitivity and reproducibility of mediastinal lymph node staging, while also reducing radiologist workload.

## Supplementary Information

Below is the link to the electronic supplementary material.

Supplementary file1 (DOCX 88.0 KB)

Supplementary file2 (DOCX 727 KB)

## Data Availability

The imaging studies and clinical data used for algorithm development are not publicly available because they contain private patient health information. Interested users may request access to these data, where institutional approvals along with signed data use agreements and/or material transfer agreements may be needed/negotiated.

## Code availability

The code will be made available on GitHub.
