# Correction to: An [18F]FDG‑PET/CT deep learning method for fully automated detection of pathological mediastinal lymph nodes in lung cancer patients

**DOI:** 10.1007/s00259-022-05855-0

**Published:** 2022-05-31

**Authors:** David Wallis, Michaël Soussan, Maxime Lacroix, Pia Akl, Clément Duboucher, Irène Buvat

**Affiliations:** 1 Laboratoire D’Imagerie Translationnelle en Oncologie, U1288 Inserm, Institut Curie, PSL, Université Paris Saclay, Paris, France; 2 Department of Nuclear Medicine, Avicenne Hospital, APHP, Bobigny, Paris, France

**Correction to: European Journal of Nuclear Medicine and Molecular Imaging**

10.1007/s00259-021-05513-x

The author regret that the author names for reference 27 of the original article are incorrectly formatted. The correct format appears below:

C Nioche, F Orlhac, S Boughdad, S Reuzé, J Goya-Outi, C Robert, C Pellot-Barakat, M Soussan, F Frouin, and I Buvat. LIFEx: a freeware for radiomic feature calculation in multimodality imaging to accelerate advances in the characterization of tumor heterogeneity. Cancer Research 2018; 78(16):4786–4789.

