# Supplementary material for: An [18F]FDG-PET/CT deep learning method for fully automated detection of pathological mediastinal lymph nodes in lung cancer patients
Source: Eur J Nucl Med Mol Imaging. 2021 Sep 14;49(3):881–8. doi: 10.1007/s00259-021-05513-x (PMC8803782; doi:10.1007/s00259-021-05513-x)

**Supplementary Material - An [18F]FDG-PET/CT deep learning method for the automated identification of pathological mediastinal lymph nodes in lung cancer patients**

David J. Wallis^1^(MSc), Michaël Soussan^2^ (MD, PhD), Maxime Lacroix^2^ (MD), Pia Akl^1^ (MD), Clément Duboucher^2^ (MD), Irène Buvat^1^ (PhD)

^1^ Laboratoire d’Imagerie Translationnelle en Oncologie, U1288 Inserm, Institut Curie, PSL, Université Paris Saclay, France; ^2^ Department of Nuclear Medicine, Avicenne Hospital, APHP, Bobigny, France

**Corresponding author:**

David Wallis

E-mail: [wallisphd@](mailto:wallisphd@)gmail.com

ORCID ID: 0000-0002-0687-4994

**SUMMARY OF PARAMETERS TESTED DURING MODEL OPTIMISATION**

A variety of network architectures and parameters were tested during optimisation of the model. As is usual when building deep learning models, these were found using educated trial-and-error. Most parameters were reasonably robust, meaning slight changes did not significantly alter the results. They are explained in detail below. All model optimisation was performed on the validation set.

**Preprocessing – Finding the Lung Region**

The whole-body scans were large, with most of the volume irrelevant to the task. We wanted to find a simple way to isolate the lung region. To do this we used a lung segmentation algorithm based on thresholding [25]. Although crude, we found this method reliably isolated the lung region on data from both scanners. It was robust enough to work on the data from the second scanner without adjustment. While a deep learning based segmentation model could be built (or incorporated into phase one), we did not have access to any segmented data, and thought this simpler method was a better option.

**Voxel Size**

Voxels were resampled so that all images had the same spatial sampling. Voxels of side length 1 mm, 1.5 mm, and 2 mm were tested. Results were marginally better at the higher resolution of 1 mm.

**PET Upper Threshold**

The vast majority of SUV values on the PET scans are within the range 0-8 SUV. However, the maximum for the entire dataset was 71 SUV. When rescaling this can give a very skewed dataset, with most values at the lower end of the scale. To resolve this problem an upper threshold was put on the PET values, with values above the threshold set to the threshold. Thresholds of 4, 8, and 12 SUV were tested. Performance was marginally better with a threshold of 8 SUV.

**CT Upper Threshold**

Similarly for the CT, upper thresholds of 400, 1000, and 2000 HU were tested. A threshold of 1000 HU gave the best performance.

**U-Net Model**

The U-Net architecture is detailed in Fig. 1. It is similar to the original U-Net architecture described in [26]. All convolutional layers have 3x3 kernels, use padding, and are followed by a ReLU activation. Some dropout was added in later layers to reduce overfitting. All downsampling (pooling) and upsampling layers have 2x2 kernels with stride 2. The final layer is a 1x1 convolution with a sigmoid activation function that maps each pixel value to a score between 0 and 1. Similar architectures with fewer filter maps per layer were also tested, but did not perform as well.

**U-Net Labelling**

No explicit segmentation for the nodes was available, so to train the U-Net spheres centred on the node coordinates were used as labels. Sphere radii of 5 mm, 10mm, and 15 mm were tested. 15 mm radii gave the best results. We found that using the smaller radii resulted in significantly poorer performance, probably because there were too few positive labels in the images. Using bigger radii would mean the spheres were much larger than the actual nodes, reducing the usefulness of phase one.

**U-Net Loss**

Cross-entropy loss, DICE loss, and Focal Tversky loss functions were tested while training the U-Net. Differences were marginal, but we found that models trained using a DICE loss were slightly more stable to changes in other parameters.

**U-Net Negative Training Slices**

When training the U-Net model, only slices with positive labels were used. This meant that the model did not ‘see’ any slices with no positive nodes during training. To test if training with negative slices improved performance, models were tested with the U-Net training set containing 5 and 10 negative slices per patient. However, we found that these models did not perform well, probably because it meant there were too few positive labels in the training images.

**Post-Phase One Removal of Small Volumes**

The aim of this step was to remove very small regions that were clearly not nodes (of the order of a few voxels). We did not systematically test different values, but removing volumes smaller than 300 voxels gave visually satisfactory results. The smallest volume containing a node in the validation set was 1800 voxels, safely above our threshold.

**Post-Phase One Dilation**

After generating a set of suspicious regions in phase one, phase two involved cutting cubes at regular intervals across these regions. To ensure that each node was contained in as many cubes as possible, we first dilated the suspicious regions. We tested models with no dilation, three-iteration, and five-iteration dilations. Three-iteration dilation models were significantly the best-performing.

**False Positive Reduction Model**

As well as the 50-layer 3D ResNet model, several custom-made models were tested. These took inspiration from other published studies that used multi-modal deep learning networks. However, we found that the ResNet model significantly outperformed these.

**Cube Size**

For input into the ResNet, cube sizes of 32 mm and 64 mm were tested. The size of cube needed to strike a balance between being big enough to contain contextual physiological information for classification, without being so big that the node itself became insignificant. We found that larger cubes of side length 64 mm performed slightly better than 32 mm.

**Node Range**

The node range is the distance that a positive node could be from the centre of a cube for the cube to be labelled as positive. For cubes of side length 64 mm, node ranges of 16 mm and 32 mm were tested. We found that using a node range of 32 mm (i.e. marking a cube as positive if it contained a node) resulted in a poorer performance than using 16 mm. This is possibly because, with a node range of 32 mm, cubes with nodes at their edges or corners would be marked as positive, despite not containing the whole node.

**Modality**

As well as [18F]FDG-PET/CT, [18F]FDG-PET-only and CT-only inputs were tested. The model architectures were adjusted accordingly (changing the number of channels from two to one). We found that using only [18F]FDG-PET or only CT significantly reduced the performance. This is not a completely fair comparison, as other parameters in the model had been optimised for [18F]FDG-PET/CT scans, but we did not pursue this investigation further.

**Learning Rate**

For both the U-Net and ResNet networks different learning rates were tested. We found that learning rate and batch size significantly affected the results. This was therefore one of the first parameters we optimised.

**Batch Size**

For the ResNet we tested batch sizes of 8, 16, and 32. We found that increasing the batch size improved the performance, with a batch size of 32 resulting in the best performance. We did not test higher batch sizes because of hardware limitations.

**Augmentation**

For both the U-Net and ResNet, random flips, rotations and translations in all axes, and operations zooming in and out, were tested as augmentation. For the U-Net we found that these did not improve the performance. For the ResNet we found that flips and rotations did slightly improve the performance.

**Transfer Learning Setup**

For transfer learning we tested learning rates of 10^-5^, 10^-6^, 10^-7^, and 10^-8^ with all layers unfrozen and fine-tuning while freezing the first 14, 24, and 34 layers with a learning rate of 10^-5^. We found small differences between the different tests (often insignificant because of the validation set size). We chose a learning rate of 10^-8^ with no freezing of layers, as this gave a marginally better performance.

**Fig. 1** U-Net architecture used in phase one. ‘Conv’ indicates convolutional layers, all with padding, 3x3 kernels, and followed by ReLU activation functions. The number indicates the number of filter maps. Max pooling and upsampling layers all had 2x2 kernels with strides of 2. For dropout layers, the number indicates the proportion of nodes ‘removed’. The final 1x1 convolutional layer is followed by a sigmoid function, which gives each output pixel a probability from 0 to 1. Horizontal arrows indicate the U-Net concatenation connections, whereby outputs from the downsampling layers are concatenated with outputs from the upsampling layers.


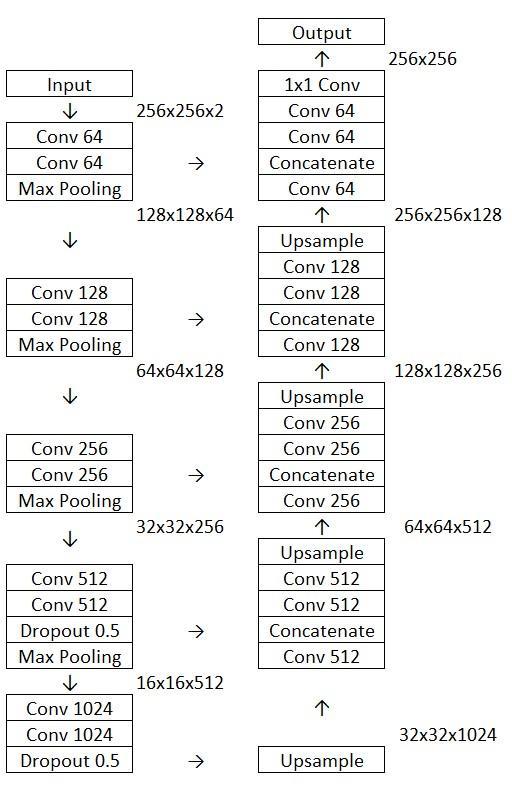

Supplement: Supplementary file 1 — Supplementary file1 (DOCX 88.0 KB) [file 259_2021_5513_MOESM1_ESM.docx]
