# Supplementary material for: An [18F]FDG-PET/CT deep learning method for fully automated detection of pathological mediastinal lymph nodes in lung cancer patients
Source: Eur J Nucl Med Mol Imaging. 2021 Sep 14;49(3):881–8. doi: 10.1007/s00259-021-05513-x (PMC8803782; doi:10.1007/s00259-021-05513-x)

**Supplementary Figure 1:** [18F]FDG-PET/CT images showing examples of true positives (a) and (b), false positives (c) and (d) and false negatives (e) and (f). The green areas show where the model predicted positive nodes. The white crosses show where the physician marked positive nodes. CT images (left column), PET images (middle column), and hybrid images (right column) are shown.
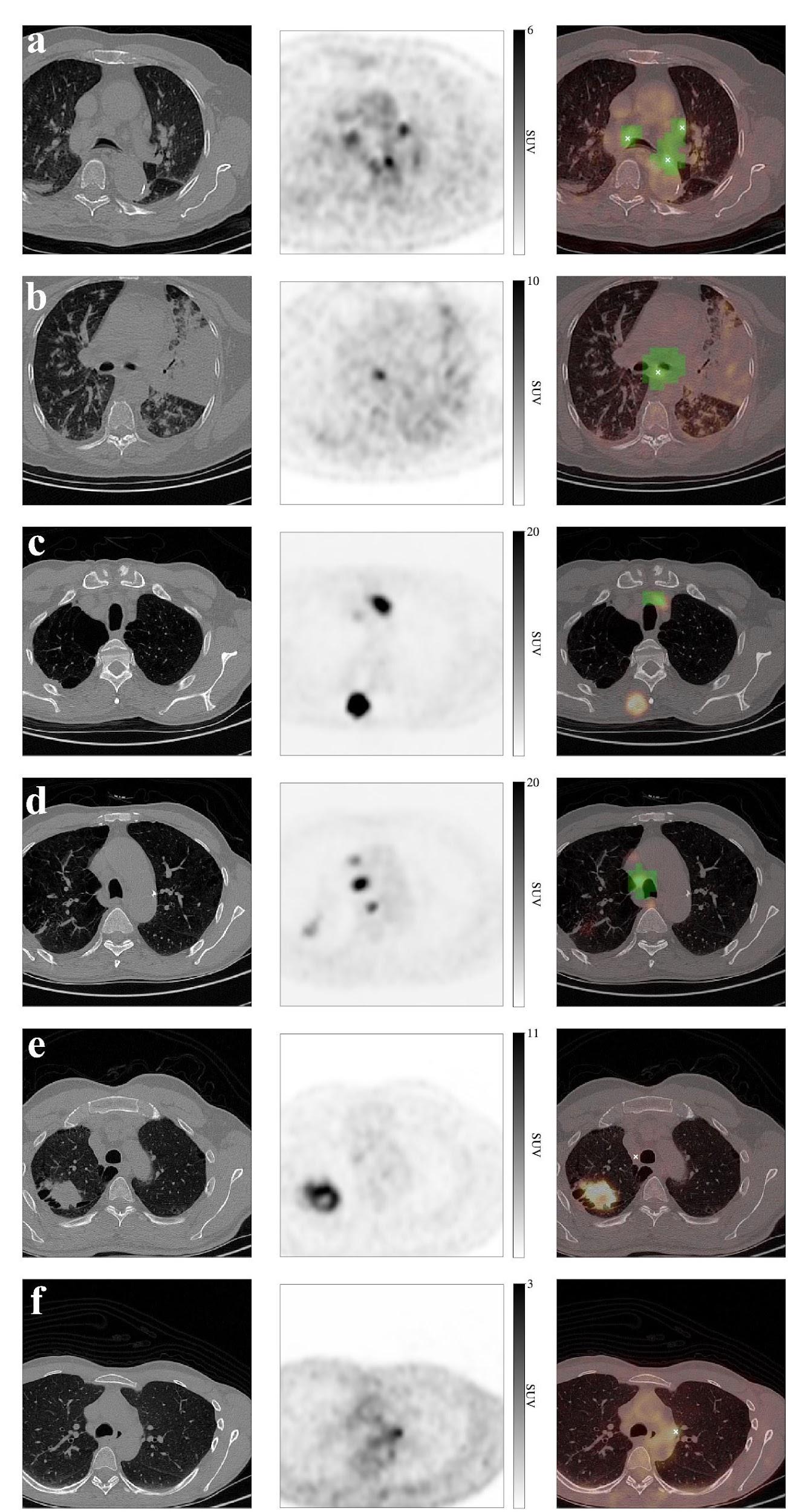


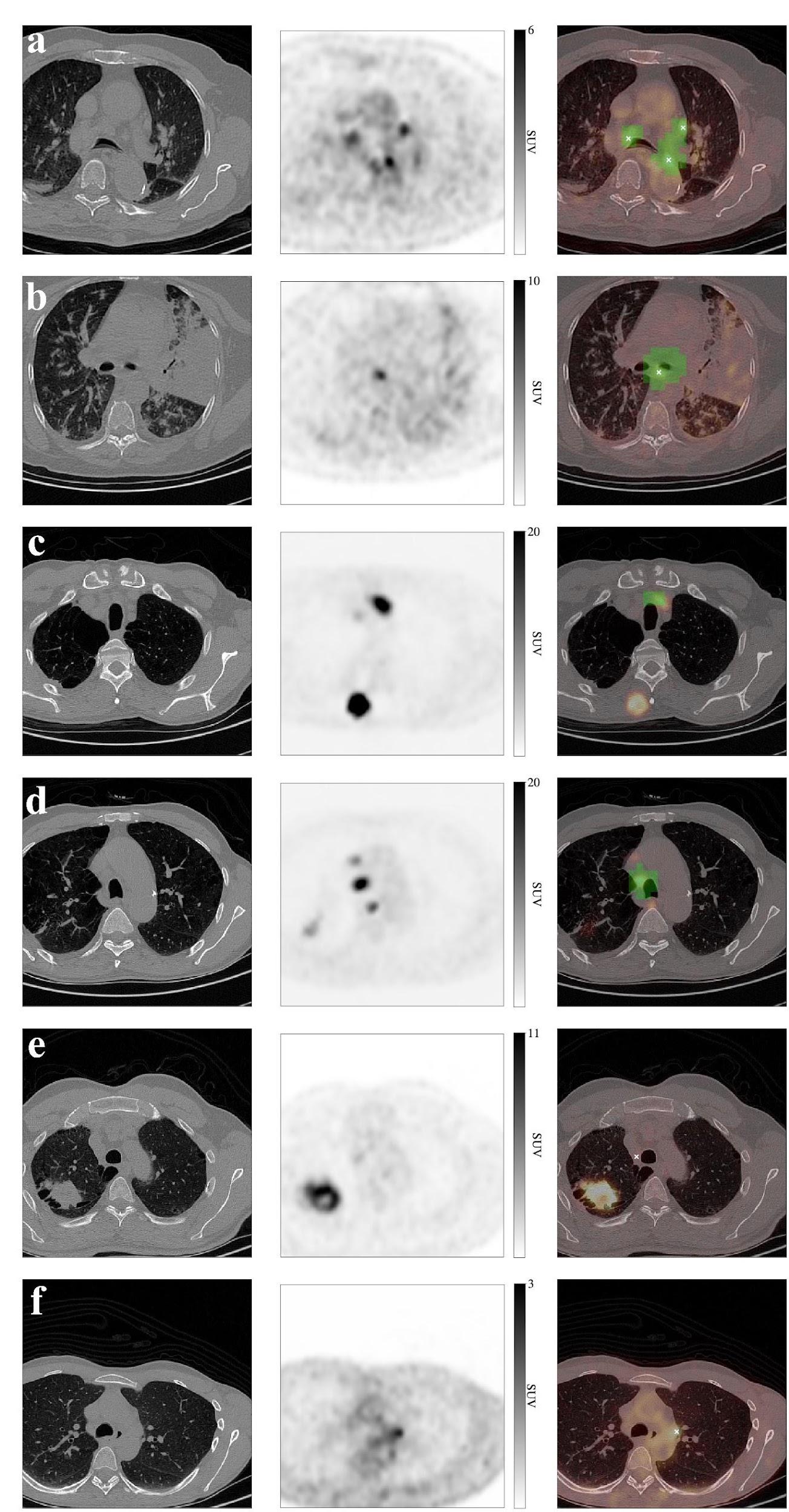

Supplement: Supplementary file 2 — Supplementary file2 (DOCX 727 KB) [file 259_2021_5513_MOESM2_ESM.docx]
